# An Approach to Using Electrical Impedance Myography Signal Sensors to Assess Morphofunctional Changes in Tissue during Muscle Contraction

**DOI:** 10.3390/bios14020076

**Published:** 2024-01-31

**Authors:** Vladislava Kapravchuk, Andrey Briko, Alexander Kobelev, Ahmad Hammoud, Sergey Shchukin

**Affiliations:** Department of Medical and Technical Information Technology, Bauman Moscow State Technical University, 105005 Moscow, Russia; briko@bmstu.ru (A.B.); ak@bmstu.ru (A.K.); hammoud@bmstu.ru (A.H.); schookin@bmstu.ru (S.S.)

**Keywords:** biosensory system, strain gauges, electrode system, electrical impedance myography, upper limbs, bionic control, skin-fat layer, laboratory stand, control system, neuromuscular interface

## Abstract

This present work is aimed at conducting fundamental and exploratory studies of the mechanisms of electrical impedance signal formation. This paper also considers morphofunctional changes in forearm tissues during the performance of basic hand actions. For this purpose, the existing research benches were modernized to conduct experiments of mapping forearm muscle activity by electrode systems on the basis of complexing the electrical impedance signals and electromyography signals and recording electrode systems’ pressing force using force transducers. Studies were carried out with the involvement of healthy volunteers in the implementation of vertical movement of the electrode system and ultrasound transducer when the subject’s upper limb was positioned in the bed of the stand while performing basic hand actions in order to identify the relationship between the morphofunctional activity of the upper limb muscles and the recorded parameters of the electro-impedance myography signal. On the basis of the results of the studies, including complex measurements of neuromuscular activity on healthy volunteers such as the signals of electro-impedance myography and pressing force, analyses of the morphofunctional changes in tissues during action performance on the basis of ultrasound and MRI studies and the factors influencing the recorded signals of electro-impedance myography are described. The results are of fundamental importance and will enable reproducible electro-impedance myography signals, which, in turn, allow improved anthropomorphic control.

## 1. Introduction

Today, the market offers a wide range of various highly functional devices for rehabilitation, from bionic prostheses (Ottobock, Touch Bionics, MaxBionic, Vincent Systems, Motility) and orthoses (MyoPro) to systems using virtual reality technology (iMotions). Separately, it is worth noting solutions in the field of exoskeletons, the use of which can be applied not only for medical but also for special issues (EduExo Pro, ReWalk, REX, Hybrid Assistive Limb, ExoAtlet). In the last two decades, upper limb exoskeletons used for maintenance and rehabilitation have received much attention from the biomedical and engineering sectors. Technology is becoming important for potential solutions for the physically weak or disabled [1], as well as for increasing the productivity of workers to perform difficult or harmful tasks [2,3].

One of the key components in the above-mentioned modern robotic devices is the bionic control module, the development of which, despite technological progress, still faces certain limitations and problems, making the final implementation far from meeting the user requirements.

In existing commercially available control systems for modern devices, the key point is the method of obtaining information about the type of actions performed by a human limb. The control methods being developed today, those using individual signals obtained on the basis of one physical method, do not allow for the obtaining of detailed information about the biomechanical characteristics of muscle activity and only partially satisfy the requirements of anthropomorphic control [4]. Thus, the high functionality and accuracy of the actions of modern actuators [5] are limited by the maximum capabilities of existing bionic control methods [6].

One of the modern promising, but requiring further study, methods of bionic control is the method of electrical impedance myography (EIM). It is known that during contraction, the specific electrical resistance of the muscle changes [7], which, in turn, makes it possible to detect the action. The use of EIM in modern commercially available systems is still limited due to the lack of physiological interpretation of EIM signal formation and the lack of high-performance equipment [8].

When recording electrical impedance, it is necessary to consider the effect of pressing the electrode system on the skin surface, which is associated with changes in the pressing force of the electrode systems [9], changes in the thickness of subcutaneous tissue [10], and the displacement of muscles relative to the location of the electrode system [11]. This fact causes difficulties in the reproducibility of measurement results and high-quality recording of signals if they are carried out without monitoring the pressing of the electrode systems to the skin surface. Inconsistency in the pressure applied to the electrode system can lead to changes in the recorded electrical impedance signal that are not related to the execution of the action. Current research does not allow us to unambiguously determine and evaluate the mechanisms of formation of the electrical impedance signal. In turn, the determination and study of the parameters underlying the signal formation mechanisms allow us to obtain new opportunities and approaches to biocontrol problems.

In turn, changes in the thickness of biological tissues and the morphology of the forearm muscles when performing hand actions can be assessed using MRI or ultrasound. It is advisable to study the probing area to determine the thickness and anatomical features of the media in the probing area at rest and when performing various types of actions to further consider the results obtained when recording the EIM signal.

In order to evaluate the effect of the changes in muscle morphology and skin-fat and muscle layer thicknesses on the electrical impedance signal during basic hand actions—as well as the effect of pinching on skin-fat and muscle layer thicknesses and internal muscle geometry (forearm anatomy)—a specially designed laboratory system is used in the present work.

## 2. Materials and Methods

### 2.1. Aspects of Using an Electrical Impedance Signal as a Control Signal

For several decades, myoelectric control of robotic upper limb prostheses has used electromyography (EMG) as a control signal to determine a person’s intentions to perform actions; however, this method has limitations and shortcomings that do not allow it to meet the requirements of anthropomorphic control. Despite the widespread use of the EMG method, both in commercially available systems and in clinical practice, it is not acceptable to combine several actions while maintaining proportional control for each. To overcome such limitations, alternative approaches are currently being widely researched and proposed. In turn, the EIM method is promising but requires further study [12].

EIM is a non-invasive method for determining neuromuscular activity, which consists of passing a high-frequency probing current between current electrodes and recording the resulting potential difference on the measuring electrodes located in the projection of the muscles of interest at rest or while performing an action [12,13].

Most applications use the tetrapolar measurement method to minimize electrode polarization, thereby maintaining the signal’s quality [14,15].

The greatest changes in the analyzed parameters when using the electrical impedance myography method occur for the frequency of probing currents lying in the range of β-dispersion [16]. For alternating currents, the maximum sensitivity of the human nervous system is approximately in the range from 10 Hz to 1 kHz and decreases at higher frequencies [17]. At low frequencies (up to 10 Hz), the electrode–skin resistance that occurs when recording biosignals has a strong influence [18]. This can lead to an imbalance at the amplifier input, which, in turn, will worsen common mode rejection [19]. Thus, the frequency of the probing current should be from 50 to 150 kHz. In accordance with international standards for the safety of medical electrical products, the effective value of the probing current that can be passed through the patient’s tissue should not exceed 100 μA∙f, where f is the operating frequency in kHz. To measure electrical impedance in the frequency range from 50 to 100 kHz, the amplitude of the probing current should not exceed 10 mA [20].

As a result of the analysis of changes in electrical impedance for each action, it was previously determined that their distinction could be made using two channels when the electrode systems are located in the projection of the radial flexor muscle of the wrist (upper channel) and ulnar extensor muscle of the wrist (lower channel). However, the use of EIM in modern commercially available systems is still limited due to the lack of physiological interpretation of EIM signal conditioning and the lack of high-performance equipment—the selection or development of the device depends on many factors, including accuracy, ease of use, cost, and the measurement range. There are various sources of error that can affect impedance and repeatability, including stray capacitance between cables, inconsistent electrode contact, electrode positioning errors, or a combination of factors [21,22,23,24]. The magnitude of the EIM signal and its change during muscle contraction/relaxation depends on the mechanisms of signal formation [8,25,26], among which we can distinguish morphological changes in muscles, the thickness of the skin-fat layer, the pressing force of the electrode system, and changes in media-conductivity-sensing areas [26,27,28,29]. None of these signal-shaping mechanisms can be achieved individually, which makes it difficult to evaluate the above contributions in practice [30,31].

### 2.2. Mechanisms of Electrical Impedance Myography Signal Formation

The literature review shows that the existing approaches do not always consider the need to select acceptable sizes of electrode systems (ES) when applying the EIM method, which is important from the severity and reproducibility of the signal point of view [32,33]. Depending on the electrode system dimensions used (inter-electrode distances), the contribution of individual signal formation mechanisms may differ, which leads to varying results of EIM studies [33].

As a result, morphological changes in muscle tissue occur, and the skin-fat layer thins during action, which, in turn, can affect the values of the electrical impedance signal [34]. Also, a change in the thickness of the skin-fat layer can occur because of an increase in the pressing force of the electrode systems on the surface of the skin, which, in turn, affects the electrode–skin resistance and the electrical impedance signal [10].

Thus, it is necessary to determine the optimal pressing force and deepening of the electrode system, as well as to evaluate the changes in the thickness of biological tissues and muscle morphology that occur because of muscle contraction, to obtain a high-quality electrical impedance signal.

### 2.3. Field of Study

In commercial prostheses, based, for example, on the surface EMG method, signals are collected by electrodes from two groups of muscles of the stump (flexors and extensors) and fed through amplifiers to the electromechanical hand control system [35]. In turn, an increase in the number of electrodes and their locations leads to an increase in the cost of such systems [36].

Prostheses at the level of the hand and fingers are designed to compensate for lost functions such as grasping and holding objects with a certain force. Prostheses at the level of the forearm and after its isolation are intended mainly for manipulation: moving objects from one position to another [35].

**Figure 1:**
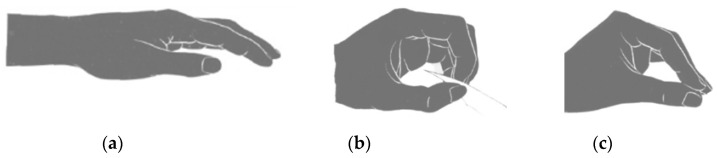
Position of the hand at rest (**a**); brush position in action state: closed “round” grip (**b**); and closed “extended” grip (**c**).

The hand takes a resting position lying on a horizontal surface (Figure 1a). The most common action of a hand for everyday use is gripping. Among the various types of grippers, two of the most popular control systems can be identified: closed “round” gripper (Figure 1b) and closed “extended” grip (Figure 1c). The position of the grip determines the grasping of an object with the index and thumb or middle and thumb. This is a closed “round” grip. The grip can be changed in such a way that the fingers extend and meet each other with wider surfaces. In this case, the terminal phalanges of the index and thumb are extended instead of flexed. This is the position called the closed extended (extended) grip position [37]. In turn, the action of flexion/extension of the hand is not implemented in most bionic devices due to technical difficulties.

**Figure 2:**
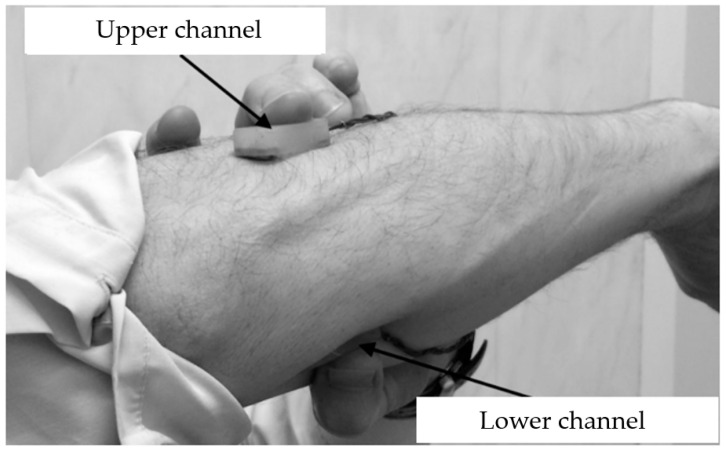
Localization of electrode systems of control channels (right hand).

Figure 2 shows the location of the measuring channels, which can be determined by palpation when performing hand actions: the upper channel is in the projection of the radial flexor muscle of the wrist and extensor digitorum, while the lower channel is in the projection of the ulnar extensor muscle of the wrist.

### 2.4. Parametric Assessment of Morphological Changes in Forearm Tissues When Performing Hand Actions Based on MRI

To study the morphological changes during actions, a series of MRI images of the forearm area, obtained from three healthy volunteers, were analyzed: women, age 22.3 ± 0.5 years; weight 57 ± 5 kg; height 168 ± 4 cm; with an average forearm circumference of 23.0 ± 1.4 cm in the upper third (mean ± SD). Axial T1-weighted MR images of the forearm (repetition time TR 1010 ms; echo time TE 11 ms) from the wrist to the elbow joint in 7.5 mm increments were acquired on a magnetic resonance imaging scanner (Magnetom Avanto, Siemens, Munich, Germany) with an induction magnetic field 1.5 Tesla.

**Figure 3:**
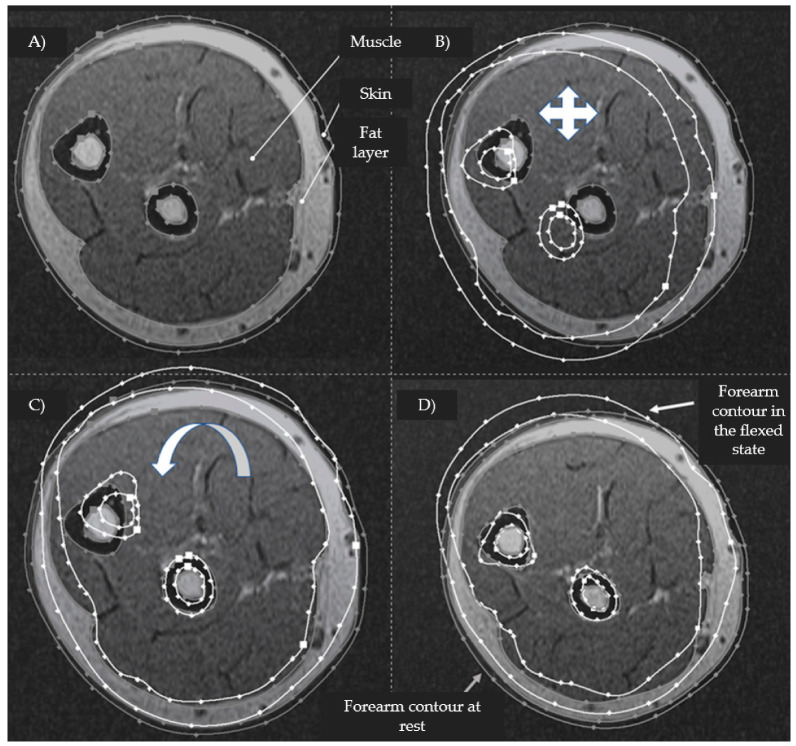
An example of contouring the skin-fat, muscle, and bone layer according to an MRI image and applying the corresponding contours (gray contour—the contour of the forearm at rest; white contour—the contour of the forearm in a state of flexion) after combining the outlines of the ulna and radius bones ((**A**)—contouring the layers using the “Spline” function; (**B**)—superimposition of the corresponding sections; (**C**)—alignment along the outlines of the radius; and (**D**)—alignment along the outlines of the ulna).

For each volunteer, studies were performed for three positions of the hand: rest, flexion, and extension. The subjects were placed in the tomograph in a prone position with their right arm raised above their head. During the research procedure, the volunteer’s forearm was fixed and did not change its position. The analysis of the obtained MRI sections of the forearms was carried out as follows: the resulting series of MRI images were converted into raster images and loaded into the Autodesk Inventor three-dimensional design system. Figure 3 shows, at the first stage of analysis of the obtained MRI images, each cross-section of the forearm, represented by the skin-fat, muscle, and bone layers, manually contoured using the “Spline” function.

To analyze the morphological changes in the forearm during action, the contours of the slices corresponding to the position of rest, flexion, and extension of the hand were superimposed in pairs and compared with each other (Figure 3B). To eliminate the artifact of forearm displacement between studies, when superimposed, the contours were combined according to the outlines of the bone structures, and if their areas differed, the slices were shifted relative to each other to find the contours whose bone outlines most accurately matched in shape, size, and relative position (Figure 3C,D).

During the work, two quantitative methods for assessing the morphological changes based on MRI sections of the forearm were studied—the method of one-dimensional sections, which is an assessment of the changes in tissue thickness in the radial direction, and the area method, which is an assessment of the changes in their areas. For analysis, sections were selected from each series related to the upper third of the forearm of volunteers—a typical location for applying the electrode system for recording the electrical impedance signal in bionic control systems, corresponding to the area of projections and the largest thickening of the finger flexor and radial flexor muscle of the wrist.

The areas of the flexor and extensor muscles were calculated for each individual section for three subjects, and the dynamics of their changes during the actions “flexion” and “extension” were determined. For each volunteer, 8 sections were analyzed from the upper third of the forearm (24 sections in total). The pattern of changes in the areas of the selected areas of the flexor and extensor muscles was determined, expressed in the proportion of slices in which this pattern was observed relative to the total number of slices. Based on the data obtained, it can be revealed that the pattern of changes in the areas of the flexor and extensor muscles during flexion varied between subjects, while the pattern corresponding to extension was the same for all volunteers. When performing flexion, in 87% of cases, the pattern of thickening of the flexors was characteristic, of which in 33% of the sections, the extensors thickened, and in 67%, they became thinner. Thus, the most characteristic pattern when performing flexion was the thickening of the flexors while thinning the extensors. When performing extension, the predominant pattern was the thickening of the extensors with a corresponding thinning of the flexors (92%).

According to the morphological changes in the forearm obtained by the method of one-dimensional sections for each individual subject, a characteristic dependence was observed in the change in the thickness of the forearm muscles active during flexion and extension; however, the resulting patterns of changes in the muscle groups turned out to be different for each of the volunteers.

Such a divergence of results may be because muscle contraction is accompanied by thickening and thinning in the transverse direction, simultaneously with a longitudinal displacement of the myogaster [38,39]. Displacement of the myogaster, as a result of contraction, was confirmed on a T2-weighted MRI image of the forearm (single excitation sequence SSFSE; echo time TE 55 ms) on a magnetic resonance imaging scanner (SIGNA Architect, GE HealthCare, Chicago, IL, USA) with a magnetic field inductance of 3 Tesla.

However, analysis of one-dimensional morphological changes in the forearm demonstrates that the thickness of the skin-fat and muscle layers do not reflect the integral volumetric morphological change in muscles. Since obtaining dynamic sections is difficult to implement using MRI, further assessment of the morphofunctional changes in the tissues of the forearm during hand actions was carried out using the ultrasound method.

In order to assess the influence of the changes in muscle morphology and the thickness of the skin-fat and muscle layer on the electrical impedance signal when performing basic hand actions, as well as the effect of pressing on the thickness of the skin-fat and muscle layers and the internal geometry of the muscles (anatomy of the forearm) and electrical impedance signal, in this work, a specially designed laboratory complex was used.

### 2.5. Laboratory Facilities Diagram

**Figure 4:**
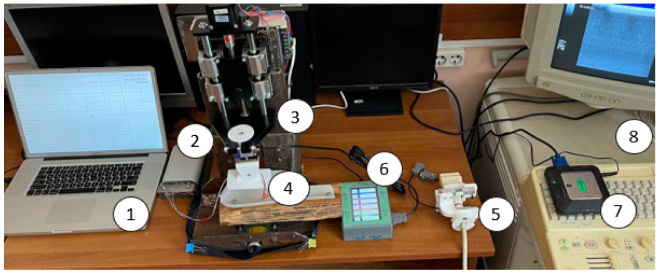
Laboratory facilities diagram (a personal computer (1), a device for bioelectric measurements “Status-A” (2), a displacement stand (3), an electrode system (4), and an ultrasonic sensor (5) were fixed in a structure with force sensors that included a device for converting mechanical signals “Stand DS” (6), a video capture card (7), and an ultrasound device (8)).

To create the precision vertical displacement of electrodes and an ultrasonic sensor under the control of pressure and vertical displacement, a laboratory facility was assembled, including a displacement stand (3), into which an electrode system (4) and an ultrasonic sensor (5) were fixed in a structure with force sensors: a device for bioelectric measurements “Status-A” (2), a device for converting mechanical signals “Stand DS” (6), an ultrasound device SIUI Apogee1100, a video capture card (7), and a personal computer (1) (Figure 4).

The facility design developed and used in the early studies of the authors [6] made it possible to ensure the fixation of the electrode system in the stand for its further positioning and displacement along three axes (along the X-axis, along the Y-axis, and along the Z-axis). The design also made it possible to simultaneously record the electrical impedance signal (using the electrode system) and the force of pressing the electrode system to the skin surface (using force sensors). The disadvantage of the described design was the distortions of the strain gauges when the electrode system was excessively pressed to the surface of the skin, as well as the preload on the sensors in the stand design, which could result in the registration of unreliable pressure force signals.

To overcome the shortcomings of the original design for ultrasonic research, a mount for the electrode system and ultrasonic sensor, as well as a force sensor, were designed, and a displacement stand was developed at the Department of Medical and Technical Information Technology, Bauman Moscow State Technical University [40].

The working stroke of the moving stand is 89 mm, the maximum moving speed is 60 mm/s, and the maximum moving error is less than 1 micron. To record the pressing force during ultrasound examinations, analog strain gauges were used. Table 1 shows the final technical characteristics of the laboratory facilities.

**Figure 5:**
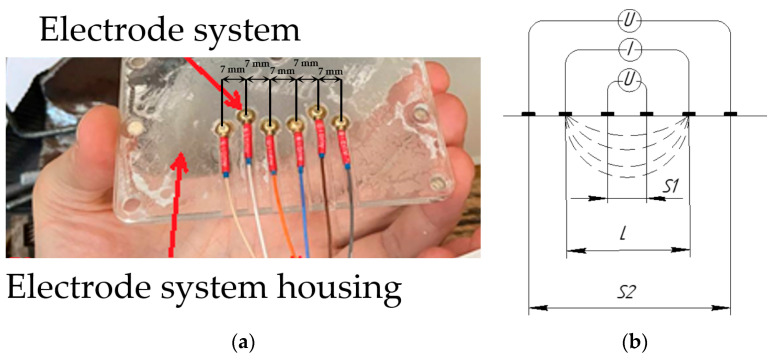
Electrode system where the red arrows indicate the parts of the system: (**a**) electrode system housing with installed reusable gold electrodes; (**b**) electrode placement scheme (S1 and S2—the distance between measuring electrodes: S1 = 7 mm, S2 = 35 mm, and L—the distance between current electrodes: L = 21 mm).

When conducting research, a two-channel electrode assembly was used, consisting of 6 electrodes located at distances of 7 mm from each other (Figure 5a). Thus, a single current channel and two independent measuring channels as part of one electrode assembly make it possible to simultaneously make 2 independent measurements using the electrode systems (Figure 5b) [3].

**Figure 6:**
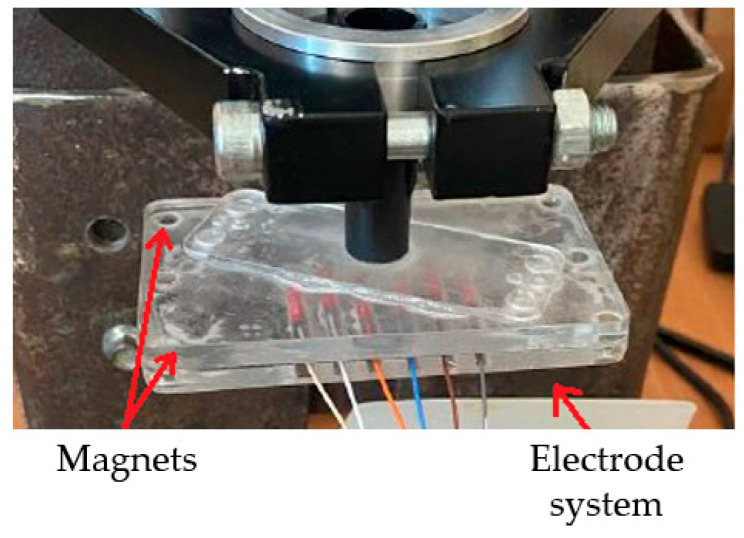
Attaching the electrode-system body to the stand for moving along the vertical axis where the red arrows indicate the parts of the system.

A conductive paste was applied to the surface of the electrode-system housing to improve electrode–skin contact. The body of the electrode system was attached to the stand using magnets (Figure 6).

**Figure 7:**
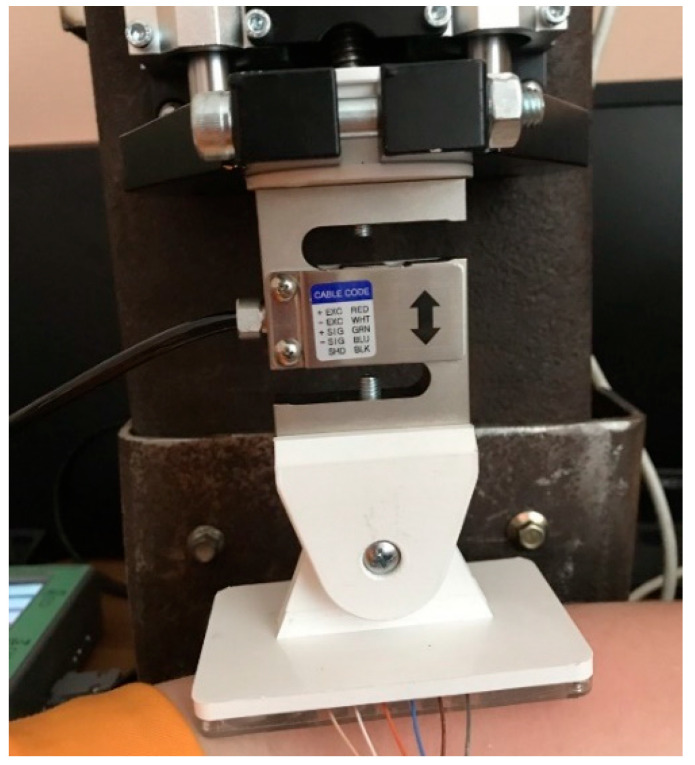
Design of attachment of the strain gauge and electrode system in the laboratory facilities.

To conduct electrical impedance studies, an analog load-measuring strain gauge sensor of the s-shaped CAS SBA 50KG, made of nickel-plated steel, was selected, the design of which will avoid distortions and incorrect recording of the pressing force when recording the EIM signal and the pressing force of the electrode system to the surface of the skin (Figure 7).

The elements for attaching the electrode system and ultrasonic sensor to the stand structure were designed, taking into account the dimensions of the displacement stand using the Autodesk Inventor program, and were printed from ABS plastic—an impact-resistant technical thermoplastic resin based on a copolymer of acrylonitrile with butadiene and styrene, the proportions of which can vary within 15–35% acrylonitrile, 5–30% butadiene, and 40–60% styrene using a Picaso Designer X Pro 3D printer.

**Figure 8:**
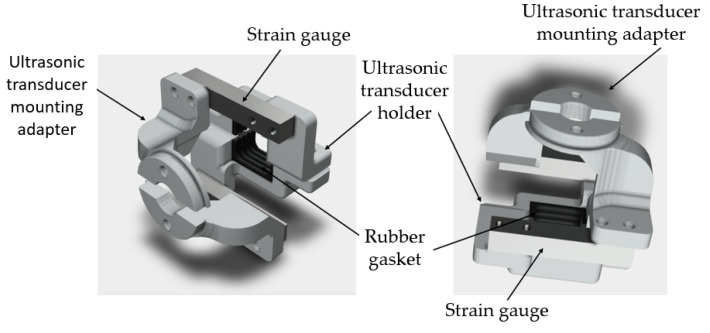
Design of attachment of the ultrasonic sensor and strain gauge.

To carry out the ultrasonic research, a mount for the ultrasonic sensor and two FSG15N1A strain gauges was designed, which also made it possible to mount the ultrasonic sensor in the stand and eliminate distortions of the strain gauge when the pressure increased (Figure 8).

### 2.6. Measurement Design

To determine the effect of pressing the electrode system onto the electrical impedance myogram and the morphofunctional changes in the tissues of the forearm when performing actions with the hand, studies were carried out on 2 volunteers (2 men aged 18–25 years, with a height of up to 185 cm, a weight of up to 110 kg, with a forearm girth of up to 32 cm). The volunteers had no previously identified or suffered injuries to the upper extremities. This work will provide data on one volunteer: gender, male; age, 34 years; height, 170 cm; weight, 100 kg; and a circumference of the forearm at the maximum thickening of the myogaster, 30.6 cm. Each volunteer signed an informed consent.

Registration of the EIM signal and ultrasound images at each step of displacement was sequentially carried out around the projection of the ulnar extensor muscle of the wrist and extensor digitorum muscles (upper channel).

The limbs of volunteers to be examined were recorded in a stand. To conduct the ultrasound, an ultrasound gel was applied to the extremity area around the projection of the ulnar extensor muscle of the wrist to record the biological signals, the skin of the study area was treated with a scrub, and electrode paste was applied to the electrodes to improve electrode–skin contact. The electrode system and ultrasound sensor, selected according to the size of the limb, were attached by means of fasteners to a structure with force sensors, which made it possible to record the pressing force.

**Figure 9:**
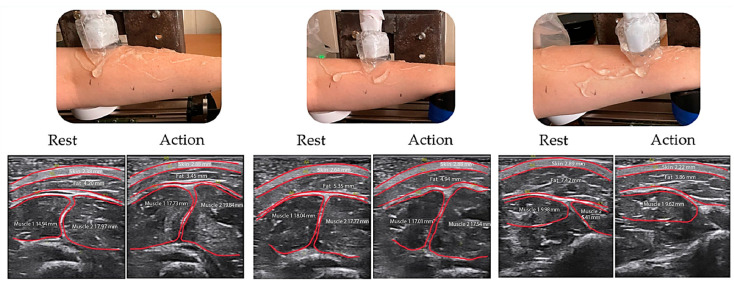
The location of the ultrasound sensor in the extreme left position, the maximum thickening of the myogaster and the extreme right position, and the corresponding ultrasound image of the anatomy of the forearm in some places and when performing an action.

Also, for each subject, transverse ultrasound images of the tissues of the forearm were recorded at rest and when performing an action in three positions of the ultrasound sensor (in the maximum thickening of the myogaster, in the extreme left and extreme right positions of the muscles in the projection of the ulnar extensor muscle of the wrist and extensor digitorum (Figure 9)).

A linear ultrasound sensor was located above the area of the muscles active during the action; before taking measurements, a conductive gel was applied to the surface of the skin. A series of measurements were carried out, during which the pressure of the ultrasound sensor on the skin surface was changed, thus simulating the increasing pressure of the electrode system during bioimpedance measurements.

Ultrasound data obtained by placing an ultrasound sensor over an active muscle group and performing an action also show that muscle contraction is accompanied by a morphological change in the muscles and a change in the thickness of the skin-fat layer, which may lead to changes in the recorded electrical impedance signal.

In order to avoid deformation of the internal structures when the ultrasonic sensor comes into contact with the skin surface, an ultrasonic gel pillow (a bag with a large amount of ultrasonic gel in which the ultrasonic sensor) was fixed.

**Figure 10:**
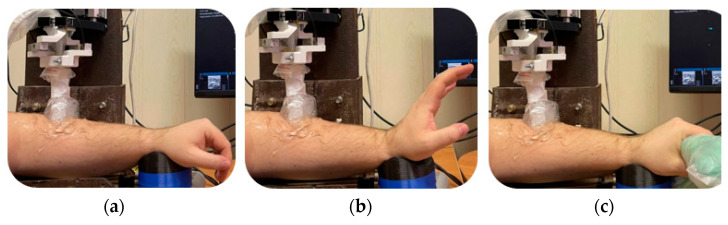
The location of the volunteer’s forearm in the laboratory bench when performing actions: (**a**) the subject’s hand in a relaxed horizontal position; (**b**) the hand is flexed at an 85-degree angle; and (**c**) the subject performs a grip.

When recording the signals, an iterative vertical displacement with a step of 1 mm was carried out on the ultrasound sensor and the electrode system vertically upward, starting with maximum pressure, which was determined by the occurrence of discomfort in the subject. At each step, the subject performed a wrist extension displacement (from the initial position to an extension position of about 85 degrees, then again to the initial position), as well as a longitudinal grip into chips (Figure 10).

Thus, at each step of displacement, EIM and ultrasound image signals were recorded in the projection of the maximum thickening of the myogaster at rest and when the subjects performed hand actions.

## 3. Results and Discussion

### 3.1. Study of Morphofunctional Activity of the Forearm Muscles When Performing Actions

**Figure 11:**
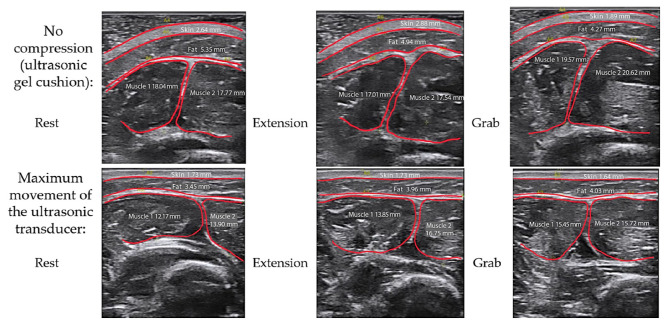
Ultrasound images of the internal anatomy of the forearm in the projection of the ulnar extensor muscle of the wrist and extensor digitorum muscles at rest, during extension of the hand and grasping without pressing and with the maximum pressing of the ultrasound sensor to the skin surface.

Figure 11 shows the ultrasound images of the internal anatomy of the forearm in the projection of the ulnar extensor muscle of the wrist and extensor digitorum muscles in the absence of compression (through an ultrasound gel pad) and with maximum pressure (displacement) of the ultrasound sensor at rest when the volunteers performed wrist extensions and grips, respectively.

**Figure 12:**
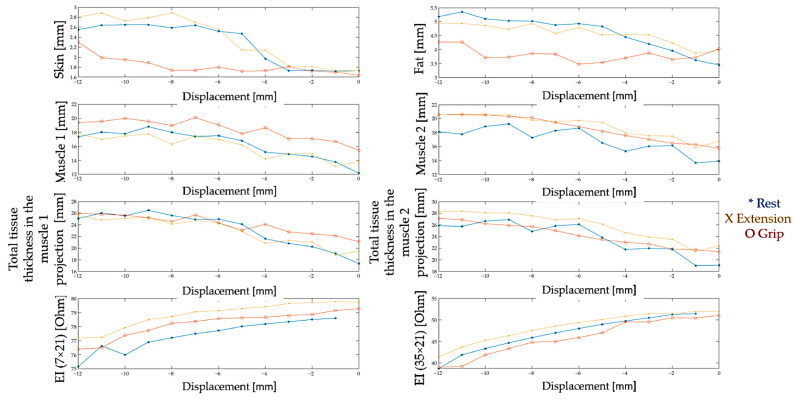
Relationship between the thickness of the skin, fat layer, and muscles and the displacement of the ultrasound sensor at rest and during actions (the blue line is a state of rest, the yellow color is extension, the red color is grip).

The ultrasound images obtained at each step of moving the ultrasound machine manually using the built-in software in the ultrasound machine were analyzed, as a result of which the dependences of the thickness of the skin, subcutaneous fat, and muscles were constructed: ulnar extensor muscle of the wrist (muscle 1) and extensor digitorum (muscle 2) on the displacement of the ultrasonic sensor (Figure 12).

It is important to keep in mind that the mechanical properties of biological tissues are individual in nature and depend on many parameters: age, diet, environmental conditions, etc. [41].

### 3.2. Study of the Amplitude Parameters of the Electrical Impedance Myography Signal at Different Pressures of the Electrode System

**Figure 13:**
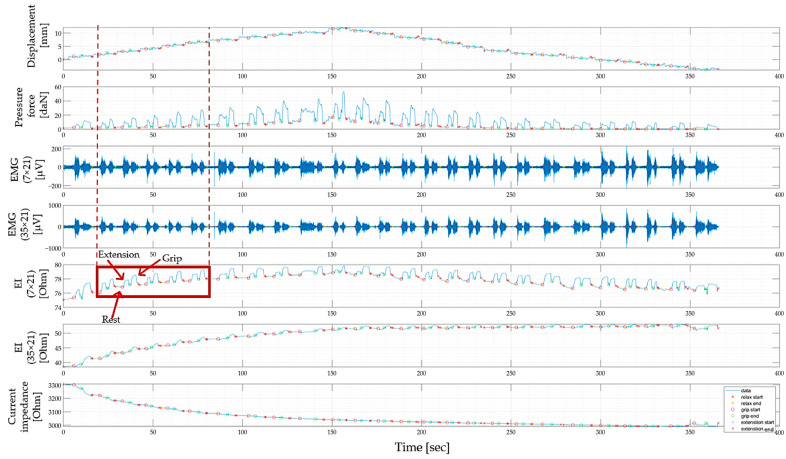
An example of signals recorded during iterative vertical displacement of an electrode system located in the projection of the ulnar extensor muscle of the wrist and extensor digitorum muscles when a volunteer performed wrist extension and pinch grip at each iteration of displacement.

Figure 13 shows an example of the biosignals from two pairs of measuring electrodes with inter-electrode distances of 7 × 21 mm and 35 × 21 mm (the scheme of electrode placement and inter-electrode distances are shown in Figure 5), as well as the registration of the signal of displacement and pressure of the electrode system to the skin surface when the sensor system is located in the projection of the ulnar extensor muscle of the wrist and extensor digitorum muscles. This occurred when a volunteer performed extension of the hand and grip at each iteration of the vertical displacement of the electrode system until maximum pressing (the appearance of discomfort in the subject and in the opposite direction to the initial position (no pressing)).

**Figure 14:**
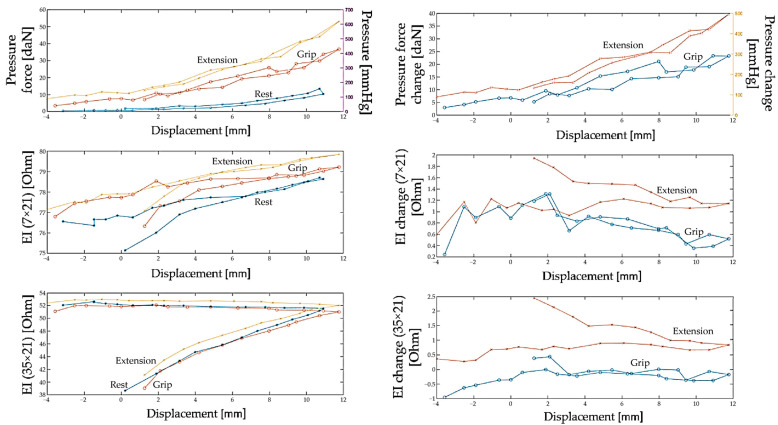
Relationship between the pressure and changes in pressure of the electrode system, the electrical impedance signal, and changes in the electrical impedance signal and displacement for inter-electrode distances of 7 and 35 mm for the resting state, as well as when the subject performs wrist extension and grip.

Also, in the current study, it is interesting to pay attention to the change in the amplitude of the EIM signal when performing an action since it is the change in the amplitude of the electrical impedance signal that can be a parameter that is analyzed to identify the relationship between the signal and morphofunctional changes in the tissues of the forearm and the types of actions performed. In Figure 14, to do this, the change in the electrical impedance signal was studied, which was calculated as the difference between the electrical resistance at rest and during action.

Based on the results obtained, we can conclude that when the electrode system is weakly pressed onto the skin surface (from 0 to 2 daN, which corresponds to a displacement of 2–3 mm), a change in the electrical impedance signal is observed when the action is performed, but the signal is unstable and cannot provide accurate proportional control.

With a further increase in the pressure of the electrode system on the skin surface (from 2–3 to 6 daN, which corresponds to a displacement of 6–7 mm), a pronounced and reproducible change in the signal is observed when performing actions, which increases the accuracy of proportional control.

A further increase in electrode system pressure (up to 10–12 daN, which corresponds to a displacement of 12–13 mm) leads to the fact that the difference between the electrical impedance at rest and during displacement sharply decreases, and it is no longer possible to detect the action.

Analysis of the data for all volunteers showed that the relationship between the change in the electrical impedance signal and the pressing (displacement) of the electrode system is repeated among volunteers; however, the values of pressing and vertical displacement at which a pronounced and reproducible pattern of the EIM signal is achieved may vary among volunteers due to various anthropometric data.

The displacement at which the thickness of the skin and subcutaneous fat remains unchanged (reaches a plateau) also varies among volunteers, depending on the anthropomorphic parameters of the volunteers and the size of the limb. Thus, after vertical displacement of the ultrasound sensor by 8–9 mm, the thickness of the subject’s skin remains unchanged at rest and when the subject performs extension and griping.

When designing a bionic control system, it is necessary to consider the relationship between the pattern of morphological changes in the forearm and the test person.

It was found that, in order to determine and study the biophysical mechanisms of signal formation, it is important to assess the morphological changes in the recording area. Thus, for biocontrol tasks, for example, in application to electrical impedance studies, it is important to study the morphological changes in muscles since this will potentially allow us to introduce new approaches to obtaining information about the displacement being performed and determining not only its type but also conducting a numerical assessment of force-moment characteristics.

The criterion for selecting the electrode location zones is maximum sensitivity and specificity to the formation of anthropomorphic neuromuscular control commands. When performing actions such as flexion–extension of the hand and grip (round and elongated), it is rational to place the electrodes on the surface of the skin directly above the antagonist muscles (in this work, the upper channel was studied—in the projection of the ulnar extensor muscle of the wrist and extensor fingers, due to convenience of the location of the limbs of the subjects in the laboratory stand and the similarity of the signal formation mechanisms for the upper and lower channels). With this arrangement of the electrode system in the upper third of the forearm, it is possible to detect the action.

To obtain a repeatable electrical impedance signal pattern associated directly with muscle contraction, it is necessary to press the electrode system to the skin by an amount determined for each user individually, which can be implemented using the approach described in this work. The pressure exerted by the electrode system on the skin and the associated nature of the changes in the boundaries of the forearm tissues during muscle contraction should be considered when developing proprioceptive control systems for bionic devices based on recording EIM signals.

This study established the influence of skin and subcutaneous fat, as well as the vertical displacement of the ultrasound sensor, on the parameters of EIM signals at rest and during hand actions. When the electrode system is pressed, the layer of subcutaneous fat becomes thinner. In this case, the skin itself stretches and, starting from certain levels of deformation, creates mechanical stresses, leading to the emergence of a counterforce to the pressing electrode.

With the aim of identifying the relationship between the parameters of the morphofunctional activity of the muscles of the upper limbs and the recorded parameters of the electrical impedance myography signal, the results of this study will allow us to take a step toward the implementation of anthropomorphic real-time control systems and copy-type control systems to create rehabilitation robotic devices and bionic manipulators. This technology can be used to create exoskeletons for medical and industrial purposes, bionic exo- and endoprostheses, and rehabilitation robotic complexes for patients with impaired motor functions with qualitatively different functionality.

## Figures and Tables

**Table 1:** Technical characteristics of the laboratory facilities.

Parameter	Result
Electrical impedance channel (EI)	
Number of measuring channels	2
Number of current channels	1
Permissible load on the current source, not less	5 kOhm
Probing current frequency	75 kHz
Signal frequency range	0–40 Hz
Measuring range	1–300 Ohm
Measurement sensitivity	10 mOhm
Motion testing facility	
Motion control unit	
Working stroke	89 mm
Maximum displacement speed	1200 rpm
Load capacity	3 kg
Maximum displacement error	1 µm
Pressure control unit	
Sensitivity	0.01 daN
Signal frequency range	0–40 Hz
Maximum pressing force	10 daN
Ultrasonic device	
Ultrasonic sensor type	Linear
Probing frequency	13 MHz
Probing depth	At least 3.9 cm
Amplification	42 dB

## Data Availability

The data presented in this study are available on request from the corresponding author.
